# 3-Fluoro-4-nitro­phenyl 4-methyl­benzene­sulfonate

**DOI:** 10.1107/S1600536811005903

**Published:** 2011-03-02

**Authors:** Wei Ang, You-Fu Luo, Yong Deng

**Affiliations:** a Key Laboratory of Drug Targeting and Drug Delivery System of the Education Ministry, Department of Medicinal Chemistry, West China School of Pharmacy, Sichuan University, Chengdu 610041, People’s Republic of China; b State Key Laboratory of Biotherapy, West China Hospital, Sichuan University, Chengdu 610041, People’s Republic of China

## Abstract

In the title compound, C_13_H_10_FNO_5_S, the dihedral angle between the benzene rings is 47.63 (14)°. In the crystal, π–π stacking occurs between nearly parallel benzene rings of adjacent mol­ecules, the centroid–centroid distance being 3.7806 (16) Å. Weak inter­molecular C—H⋯O hydrogen bonding is also present in the crystal structure.

## Related literature

For related compounds and their biological activity, see: Cho *et al.* (2003) ▶; Marson *et al.* (2007 ▶).
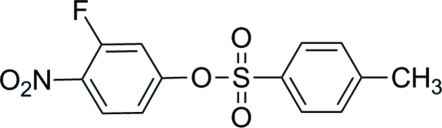


## Experimental

### 

#### Crystal data

C_13_H_10_FNO_5_S
*M* *_r_* = 311.28
Orthorhombic, 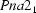

*a* = 14.2596 (5) Å
*b* = 11.4800 (3) Å
*c* = 8.3602 (2) Å
*V* = 1368.57 (7) Å^3^
*Z* = 4
Mo *K*α radiation
μ = 0.27 mm^−1^
*T* = 293 K
0.30 × 0.30 × 0.20 mm

#### Data collection

Oxford Diffraction Xcalibur Eos diffractometer
Absorption correction: multi-scan (*CrysAlis PRO*; Oxford Diffraction, 2006 ▶) *T*_min_ = 0.979, *T*_max_ = 1.0
10802 measured reflections
2251 independent reflections
1855 reflections with *I* > 2σ(*I*)
*R*_int_ = 0.025

#### Refinement

*R*[*F* ^2^ > 2σ(*F* ^2^)] = 0.034
*wR*(*F* ^2^) = 0.083
*S* = 1.05
2251 reflections
191 parameters
1 restraint
H-atom parameters constrained
Δρ_max_ = 0.12 e Å^−3^
Δρ_min_ = −0.23 e Å^−3^
Absolute structure: Flack (1983 ▶), 752 Friedel pairs
Flack parameter: −0.06 (9)

### 

Data collection: *CrysAlis PRO* (Oxford Diffraction, 2006 ▶); cell refinement: *CrysAlis PRO*; data reduction: *CrysAlis PRO*; program(s) used to solve structure: *SHELXTL* (Sheldrick, 2008 ▶); program(s) used to refine structure: *SHELXTL*; molecular graphics: *OLEX2* (Dolomanov *et al.*, 2009 ▶); software used to prepare material for publication: *OLEX2*.

## Supplementary Material

Crystal structure: contains datablocks I, global. DOI: 10.1107/S1600536811005903/xu5126sup1.cif

Structure factors: contains datablocks I. DOI: 10.1107/S1600536811005903/xu5126Isup2.hkl

Additional supplementary materials:  crystallographic information; 3D view; checkCIF report

## Figures and Tables

**Table 1:** Hydrogen-bond geometry (Å, °)

*D*—H⋯*A*	*D*—H	H⋯*A*	*D*⋯*A*	*D*—H⋯*A*
C6—H6⋯O2^i^	0.93	2.55	3.224 (4)	129

Symmetry code: (i) 
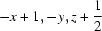
.

**Fig. 1.**
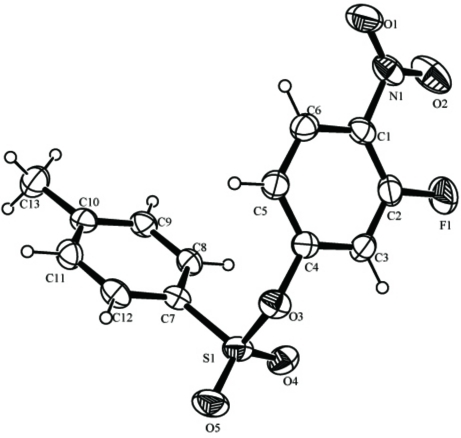
The molecular structure of the title compound, with displacement ellipsoids drawn at the 30% probability level.

**Fig. 2.**
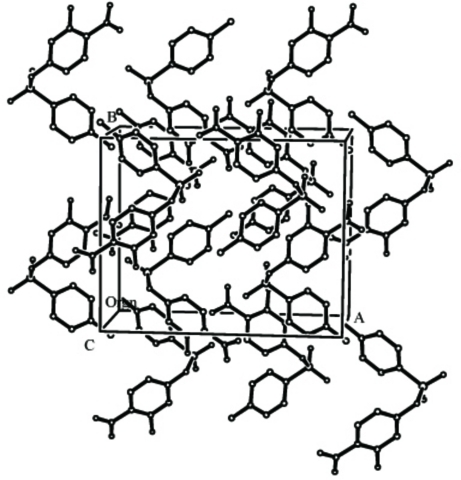
The cell packing of the title compound.

## supplementary crystallographic information

### Comment

Aryloxyalkanoic acid hydroxyamides are potent inhibitors of histone deacetylase (Marson *et al.*, 2007; Cho *et al.*, 2003). 3-Fluoro-4-nitrophenyl 4-methylbenzenesulfonate is one of the key intermediates to synthesize the aryloxyalkanoic acid hydroxyamides derivatives. We report here its crystal structure. In the title compound (Fig. 1), the dihedral angle between the 3-fluoro-4-nitrophenyl ring and the 4-methylbenzene ring is 47.63 (14)°. In the crystal, intermolecular π-π stacking [centroid–centroid distance = 3.7806 (16) Å] stabilizes the structure (Fig. 2). Weak C—H···O hydrogen bonding is present in the crystal structure (Table 1).

### Experimental

To the 3-fluoro-4-nitrophenol (19.10 mmol) in chloroform (20 ml) at 273 K were added pyridine (3.70 ml, 45.84 mmol) dropwise over a period of 20 min and *p*-toluenesulfonyl chloride (22.92 mmol) in small portions. This reaction mixture was stirred at room temperature for 12 h and diluted with dichloromethane and then 10% aqueous HCl. The separated organic layer was washed with 10% aqueous HCl, water and saturated aqueous NaCl; dried over NaSO4; and concentrated *in vacuo*. The crude 3-fluoro-4-nitrophenyl 4-methylbenzenesulfonate were purified by recrystallization. Crystals suitable for X-ray analysis were obtained by slow evaporation from a solution of ethanol.

### Refinement

H atoms were positioned geometrically and refined using a riding model with C—H = 0.93 Å and *U*_iso_(H) = 1.2U_eq_(C) for aromatic H atoms and C—H = 0.96 Å and *U*_iso_(H) = 1.5U_eq_(C) for methyl H atoms.

### Crystal data

**Table:**

C_13_H_10_FNO_5_S	*F*(000) = 640
*M**_r_* = 311.28	*D*_x_ = 1.511 Mg m^−^^3^
Orthorhombic, *P**n**a*2_1_	Mo *K*α radiation, λ = 0.7107 Å
Hall symbol: P 2c -2n	Cell parameters from 4234 reflections
*a* = 14.2596 (5) Å	θ = 2.9–29.1°
*b* = 11.4800 (3) Å	µ = 0.27 mm^−^^1^
*c* = 8.3602 (2) Å	*T* = 293 K
*V* = 1368.57 (7) Å^3^	Block, colorless
*Z* = 4	0.30 × 0.30 × 0.20 mm

### Data collection

**Table:**

Oxford Diffraction Xcalibur Eos diffractometer	2251 independent reflections
Radiation source: fine-focus sealed tube	1855 reflections with *I* > 2σ(*I*)
graphite	*R*_int_ = 0.025
Detector resolution: 16.0874 pixels mm^-1^	θ_max_ = 26.4°, θ_min_ = 2.9°
ω scans	*h* = −17→17
Absorption correction: multi-scan (*CrysAlis PRO*; Oxford Diffraction, 2006)	*k* = −14→14
*T*_min_ = 0.979, *T*_max_ = 1.0	*l* = −7→10
10802 measured reflections	

### Refinement

**Table:**

Refinement on *F*^2^	Secondary atom site location: difference Fourier map
Least-squares matrix: full	Hydrogen site location: inferred from neighbouring sites
*R*[*F*^2^ > 2σ(*F*^2^)] = 0.034	H-atom parameters constrained
*wR*(*F*^2^) = 0.083	*w* = 1/[σ^2^(*F*_o_^2^) + (0.039*P*)^2^ + 0.1799*P*] where *P* = (*F*_o_^2^ + 2*F*_c_^2^)/3
*S* = 1.05	(Δ/σ)_max_ = 0.025
2251 reflections	Δρ_max_ = 0.12 e Å^−^^3^
191 parameters	Δρ_min_ = −0.22 e Å^−^^3^
1 restraint	Absolute structure: Flack (1983), 752 Friedel pairs
Primary atom site location: structure-invariant direct methods	Flack parameter: −0.06 (9)

### Special details

**Table:**

Geometry. All e.s.d.'s (except the e.s.d. in the dihedral angle between two l.s. planes) are estimated using the full covariance matrix. The cell e.s.d.'s are taken into account individually in the estimation of e.s.d.'s in distances, angles and torsion angles; correlations between e.s.d.'s in cell parameters are only used when they are defined by crystal symmetry. An approximate (isotropic) treatment of cell e.s.d.'s is used for estimating e.s.d.'s involving l.s. planes.
Refinement. Refinement of *F*^2^ against ALL reflections. The weighted *R*-factor *wR* and goodness of fit *S* are based on *F*^2^, conventional *R*-factors *R* are based on *F*, with *F* set to zero for negative *F*^2^. The threshold expression of *F*^2^ > σ(*F*^2^) is used only for calculating *R*-factors(gt) *etc*. and is not relevant to the choice of reflections for refinement. *R*-factors based on *F*^2^ are statistically about twice as large as those based on *F*, and *R*- factors based on ALL data will be even larger.

### Fractional atomic coordinates and isotropic or equivalent isotropic displacement parameters (Å^2^)

**Table:**

	*x*	*y*	*z*	*U*_iso_*/*U*_eq_	
S1	0.16636 (4)	0.27076 (5)	0.58998 (10)	0.05567 (19)	
F1	0.34171 (15)	−0.14722 (14)	0.6292 (3)	0.1007 (7)	
O1	0.56870 (17)	−0.0441 (2)	0.8899 (4)	0.0906 (8)	
O2	0.51366 (19)	−0.1649 (2)	0.7187 (3)	0.1041 (9)	
O3	0.18775 (13)	0.20401 (17)	0.7566 (2)	0.0574 (5)	
O4	0.16341 (14)	0.18606 (19)	0.4665 (3)	0.0675 (6)	
O5	0.08578 (12)	0.33777 (18)	0.6260 (3)	0.0788 (7)	
N1	0.5080 (2)	−0.0778 (2)	0.7989 (3)	0.0675 (7)	
C1	0.4217 (2)	−0.0077 (2)	0.7863 (4)	0.0523 (7)	
C2	0.3442 (2)	−0.0442 (2)	0.7026 (4)	0.0601 (8)	
C3	0.2660 (2)	0.0248 (2)	0.6906 (4)	0.0580 (7)	
H3	0.2136	−0.0003	0.6341	0.070*	
C4	0.26682 (18)	0.1317 (2)	0.7636 (3)	0.0474 (6)	
C5	0.3427 (2)	0.1698 (2)	0.8491 (4)	0.0548 (7)	
H5	0.3417	0.2424	0.8987	0.066*	
C6	0.4204 (2)	0.0995 (2)	0.8609 (4)	0.0566 (7)	
H6	0.4722	0.1243	0.9192	0.068*	
C7	0.26414 (17)	0.3597 (2)	0.5642 (3)	0.0468 (6)	
C8	0.33831 (18)	0.3209 (2)	0.4704 (4)	0.0532 (7)	
H8	0.3359	0.2483	0.4213	0.064*	
C9	0.41530 (18)	0.3917 (2)	0.4512 (4)	0.0538 (7)	
H9	0.4659	0.3650	0.3914	0.065*	
C10	0.41940 (19)	0.5008 (2)	0.5179 (3)	0.0519 (7)	
C11	0.34570 (19)	0.5373 (2)	0.6128 (4)	0.0584 (7)	
H11	0.3483	0.6103	0.6609	0.070*	
C12	0.26858 (19)	0.4679 (2)	0.6375 (3)	0.0551 (7)	
H12	0.2198	0.4931	0.7028	0.066*	
C13	0.5028 (2)	0.5786 (3)	0.4867 (5)	0.0752 (9)	
H13A	0.4860	0.6370	0.4096	0.113*	
H13B	0.5537	0.5327	0.4460	0.113*	
H13C	0.5215	0.6155	0.5847	0.113*	

### Atomic displacement parameters (Å^2^)

**Table:**

	*U*^11^	*U*^22^	*U*^33^	*U*^12^	*U*^13^	*U*^23^
S1	0.0420 (3)	0.0668 (4)	0.0582 (4)	0.0045 (3)	−0.0005 (4)	0.0083 (4)
F1	0.1299 (17)	0.0508 (9)	0.1214 (18)	0.0063 (10)	−0.0321 (16)	−0.0197 (11)
O1	0.0640 (15)	0.0999 (18)	0.108 (2)	0.0104 (14)	−0.0161 (15)	0.0259 (17)
O2	0.135 (2)	0.0934 (17)	0.0836 (18)	0.0580 (16)	−0.0025 (17)	−0.0028 (15)
O3	0.0489 (11)	0.0689 (12)	0.0543 (12)	0.0047 (9)	0.0104 (10)	0.0087 (10)
O4	0.0635 (14)	0.0765 (13)	0.0625 (13)	−0.0099 (10)	−0.0104 (11)	−0.0038 (11)
O5	0.0452 (11)	0.0901 (14)	0.1011 (19)	0.0158 (9)	0.0081 (13)	0.0152 (14)
N1	0.0737 (19)	0.0709 (17)	0.0579 (17)	0.0151 (14)	0.0075 (15)	0.0231 (15)
C1	0.0533 (18)	0.0547 (16)	0.0487 (17)	0.0054 (12)	0.0020 (14)	0.0132 (14)
C2	0.078 (2)	0.0415 (14)	0.0605 (19)	−0.0031 (14)	−0.0059 (17)	0.0043 (14)
C3	0.0585 (17)	0.0529 (15)	0.0627 (19)	−0.0129 (13)	−0.0115 (15)	0.0035 (14)
C4	0.0457 (14)	0.0534 (14)	0.0430 (15)	−0.0017 (11)	0.0040 (13)	0.0085 (12)
C5	0.0580 (17)	0.0520 (15)	0.0543 (17)	−0.0027 (13)	−0.0022 (15)	0.0005 (13)
C6	0.0557 (18)	0.0606 (17)	0.0534 (18)	−0.0076 (14)	−0.0060 (15)	0.0069 (14)
C7	0.0431 (13)	0.0520 (13)	0.0453 (17)	0.0104 (10)	0.0012 (13)	0.0046 (13)
C8	0.0524 (16)	0.0467 (13)	0.0604 (17)	0.0088 (12)	0.0042 (15)	−0.0067 (13)
C9	0.0453 (15)	0.0563 (16)	0.0597 (18)	0.0090 (12)	0.0078 (14)	−0.0005 (14)
C10	0.0506 (16)	0.0555 (16)	0.0497 (17)	0.0051 (12)	−0.0056 (13)	0.0021 (12)
C11	0.0692 (18)	0.0491 (13)	0.0568 (18)	0.0042 (12)	−0.0026 (18)	−0.0081 (14)
C12	0.0601 (17)	0.0582 (15)	0.0471 (17)	0.0176 (12)	0.0093 (14)	−0.0015 (13)
C13	0.069 (2)	0.0714 (19)	0.085 (2)	−0.0119 (15)	0.001 (2)	−0.0069 (18)

### Geometric parameters (Å, °)

**Table:**

S1—O3	1.619 (2)	C5—C6	1.374 (4)
S1—O4	1.419 (2)	C6—H6	0.9300
S1—O5	1.4152 (19)	C7—C8	1.390 (4)
S1—C7	1.741 (3)	C7—C12	1.386 (3)
F1—C2	1.332 (3)	C8—H8	0.9300
O1—N1	1.216 (4)	C8—C9	1.375 (3)
O2—N1	1.206 (3)	C9—H9	0.9300
O3—C4	1.401 (3)	C9—C10	1.373 (4)
N1—C1	1.475 (4)	C10—C11	1.382 (4)
C1—C2	1.374 (4)	C10—C13	1.509 (4)
C1—C6	1.380 (4)	C11—H11	0.9300
C2—C3	1.372 (4)	C11—C12	1.374 (4)
C3—H3	0.9300	C12—H12	0.9300
C3—C4	1.371 (4)	C13—H13A	0.9600
C4—C5	1.369 (4)	C13—H13B	0.9600
C5—H5	0.9300	C13—H13C	0.9600
			
F1—C2—C1	121.8 (3)	C6—C1—N1	117.8 (3)
F1—C2—C3	117.2 (3)	C6—C5—H5	120.4
O1—N1—C1	117.7 (3)	C7—C8—H8	120.5
O2—N1—O1	124.3 (3)	C7—C12—H12	120.3
O2—N1—C1	118.0 (3)	C8—C7—S1	119.45 (19)
O3—S1—C7	103.48 (11)	C8—C9—H9	119.1
O4—S1—O3	107.89 (11)	C9—C8—C7	119.0 (2)
O4—S1—C7	109.59 (13)	C9—C8—H8	120.5
O5—S1—O3	103.14 (14)	C9—C10—C11	118.5 (2)
O5—S1—O4	120.23 (14)	C9—C10—C13	120.2 (3)
O5—S1—C7	110.97 (11)	C10—C9—C8	121.7 (2)
C1—C6—H6	119.9	C10—C9—H9	119.1
C2—C1—N1	122.7 (3)	C10—C11—H11	119.4
C2—C1—C6	119.5 (3)	C10—C13—H13A	109.5
C2—C3—H3	120.7	C10—C13—H13B	109.5
C3—C2—C1	121.0 (3)	C10—C13—H13C	109.5
C3—C4—O3	120.3 (2)	C11—C10—C13	121.3 (3)
C4—O3—S1	117.93 (16)	C11—C12—C7	119.3 (2)
C4—C3—C2	118.6 (3)	C11—C12—H12	120.3
C4—C3—H3	120.7	C12—C7—S1	120.46 (19)
C4—C5—H5	120.4	C12—C7—C8	120.1 (2)
C4—C5—C6	119.1 (3)	C12—C11—C10	121.3 (2)
C5—C4—O3	118.0 (2)	C12—C11—H11	119.4
C5—C4—C3	121.7 (2)	H13A—C13—H13B	109.5
C5—C6—C1	120.2 (3)	H13A—C13—H13C	109.5
C5—C6—H6	119.9	H13B—C13—H13C	109.5

### Hydrogen-bond geometry (Å, °)

**Table:**

*D*—H···*A*	*D*—H	H···*A*	*D*···*A*	*D*—H···*A*
C6—H6···O2^i^	0.93	2.55	3.224 (4)	129

Symmetry codes: (i) −*x*+1, −*y*, *z*+1/2.
